# Clinical impact and public health challenges of a PVL-MRSA bacteraemia outbreak amongst people who inject drugs in South Yorkshire, UK

**DOI:** 10.1099/acmi.0.000642.v3

**Published:** 2024-02-08

**Authors:** Matthew Beaumont, Bala Subramanian, Ken Agwuh, Yan Ryan, Bruno Pichon

**Affiliations:** ^1^​ Doncaster and Bassetlaw Teaching Hospitals, Doncaster Royal Infirmary, Armthorpe Road, Doncaster, DN2 5LT, UK; ^2^​ Sheffield Teaching Hospitals, NHS Foundation Trust, Northern General Hospital, Herries Road, Sheffield, S5 7AU, UK; ^3^​ Staphylococcal and Streptococcus Reference Laboratory, United Kingdom Health Security Agency, 61 Colindale Avenue, London, NW9 5EQ, UK

**Keywords:** Panton–Valentine leukocidin (PVL), methicillin-resistant *Staphylococcus aureus *(MRSA), people who inject drugs (PWID), outbreak, *Staphylococcus aureus*

## Abstract

**Background.:**

Panton–Valentine leukocidin (PVL) *Staphylococcus aureus* (SA) is an emergent public health concern. PVL toxin has been mostly associated with methicillin-sensitive *S. aureus* (MSSA)-related skin and soft tissue infections occurring in high-risk groups such as people who inject drugs (PWID). The emergence of PVL methicillin-resistant *S. aureus* (MRSA) infection is causing severe and life-threatening disease in PWID.

**Clinical cases.:**

We present an outbreak of eight PVL-MRSA bacteraemia cases at a UK teaching hospital between 2018 and 2022. An additional four patients developed bacteraemia with PVL-negative MRSA of the same multilocus sequence type (MLST). All patients were PWID and aged 33–51 years old. Four patients developed MRSA bacterial endocarditis. Three patients died. These cases represent the initial cases detected at Doncaster and Bassetlaw Teaching Hospitals of what is an ongoing and developing outbreak.

**Management.:**

An outbreak investigation has been undertaken in association with the UK Health Security Agency. Epidemiological factors have been explored, including via direct contact at a local sheltered accommodation and the possibility of a contaminated drug supply. Whole-genome sequencing confirmed that all isolates were closely related and of the same MLST (sequence type 5). A community substance misuse group disseminated health education on the prevention of PVL-MRSA. Preventing infection in PWID presents a major challenge due to the impact of addiction on engagement with services and the significant barriers faced by our patients in observing infection prevention measures.

**Conclusion.:**

PVL-MRSA is of major public health concern and outbreak investigation and mapping out local epidemiological patterns plays a vital role in preventing further spread throughout the community. Additionally, this work enables targeted and early treatment in patients in high-risk categories for disease. These cases of PVL-MRSA infection in PWID highlights the transmissibility, pathogenic potential and severe clinical disease spectrum within this population. Further work is required to tackle transmission and infection from this pathogenic strain.

## Data Summary

The accession number for the methicillin-resistant *Staphylococcus aureus* (MRSA) isolates presented is PRJEB62493. All sequencing data are publicly available from the European Nucleotide Archive (ENA).

**Impact Statement**

This paper highlights the pathogenic potential of Panton–Valentine leukocidin (PVL) methicillin-resistant *Staphylococcus aureus* (MRSA) in an outbreak amongst people who inject drugs (PWID). These cases highlight the severe clinical disease spectrum amongst PWID and the challenges of preventing infection in this vulnerable population.

## Introduction

Panton–Valentine leukocidin (PVL) *Staphylococcus aureus* (SA) is a major public health concern [1]. PVL toxin has been classically associated with methicillin-sensitive *S. aureus* (MSSA) related to skin and soft tissue infections (SSTIs) and much more rarely causes severe invasive disease [1]. PVL is a pore-forming exo-toxin produced by certain strains of *S. aureus* [2]. The toxin is encoded by two genes (*LukS*-PV and *LukF*-PV) and creates a pore-forming heptamer that causes neutrophil lysis and tissue necrosis [2]. There is much debate around its role as a virulence factor [1]. PVL has been shown to be a risk factor for mortality in people over 3 years old with severe community-acquired staphylococcal pneumonia [3].

People at risk for infection with PVL-SA are groups in close contact or crowded conditions, those sharing contaminated items (towels, needles) or with poor hygiene and compromised skin integrity. This includes prisoners, sports teams and people who inject drugs (PWID) [1].

Estimates of PVL-SA prevalence vary. The prevalence of PVL-producing *S. aureus* isolates has previously been suggested to be <2 % [1]. Subsequent research has detected the PVL gene in 20 % of *S. aureus* isolates from skin and soft tissue infections [4, 5]. It is unclear whether higher prevalence may be influenced by improved recognition or testing.

PVL methicillin-resistant *S. aureus* (MRSA) is an emergent public health concern [1]. PVL-MRSA is less prevalent than PVL-MSSA in the UK [4, 6], although this organism has been associated with increased morbidity and mortality in invasive infections [1]. Community-acquired PVL-MRSA has become an endemic problem in the USA, with the rise of the USA 300 clone causing nosocomial outbreaks [1, 2].

In the UK, PVL-MRSA has been associated with outbreaks amongst PWID [7]. Risk factors for colonization with MRSA amongst PWID in previous outbreaks have included frequently injecting in public places, injecting in groups of three or more, or injection site skin and soft tissue infection [7]. Outbreaks of PVL-MRSA amongst PWID have been associated with severe morbidity and mortality [7].

During 2020 an increased number of PVL- MRSA bacteraemia cases were identified at Doncaster and Bassetlaw Teaching Hospitals amongst PWID through the post-infection review process. Work with United Kingdom Health Security Agency (UKHSA) has helped to identify what is an ongoing outbreak of MRSA. UKHSA are leading on a case–control study to investigate further any common exposures or risk factors with a view to interrupting ongoing transmission. This paper represents a summary of the initial cases detected in Doncaster of what is an ongoing and developing outbreak.

## Methods

A retrospective analysis was undertaken of all MRSA bacteraemia cases isolated at Doncaster Royal Infirmary between July 2018 and July 2022. This included review of the patients’ case notes and electronic medical records noting the clinical disease spectrum and documentation of injecting drug misuse.

All MRSA bacteraemia cases were first isolated by the processing of blood culture specimens at the local microbiology laboratory at Doncaster Royal Infirmary. MRSA isolates were sent to the Staphylococcal and Streptococcus Reference Laboratory (UKHSA) for PVL testing and whole-genome sequencing (WGS).

PVL testing was performed using a real-time quadruplex PCR assay to detect the *lukS*-PV gene encoding the PVL toxin [8]. WGS DNA extraction was performed as described by Utsi *et al*. previously [9]. Genetic relatedness was confirmed by single-nucleotide polymorphism (SNP) distance thresholds, with a cutoff of 10 SNPs or fewer. Snapper DB software was used perform SNP distance threshold analysis [10]. All sequencing data are available in the European Nucleotide Archive (ENA) under accession number: PRJEB62493.

Comparison of MRSA isolates from Doncaster and Bassetlaw Teaching Hospitals was undertaken with reference to the national reference laboratory WGS archive and geographical discernment was noted.

Post-infection review was undertaken for all the MRSA bacteraemia cases. Outbreak investigation was initiated in collaboration with UKHSA to help delineate the extent of the problem and to identify risk factors associated with PVL-MRSA infection and/or colonization. Clinical management of the cases was advised on by the local microbiology department and decolonization was offered to all primary cases where appropriate. Interim guidance was issued on initial management of PWID presenting to secondary care with sepsis. The local health protection team undertook community screening and decolonization at a local sheltered accommodation and provided targeted health education at a substance misuse group.

Epidemiological research is currently being undertaken by UKHSA in partnership with stakeholders in Doncaster to review the prevalence and risk factors associated with PVL-MRSA infection or colonization amongst PWID with a view to tackling the root cause of the problem.

## Results

We detected 12 related MRSA bacteraemia cases at Doncaster Royal Infirmary between July 2018 and July 2022. All patients reported injecting drug misuse. The age range was 33–51 years old (median age: 41) (Table 1). There were nine male and three female patients. A total of eight PVL-positive MRSA bacteraemia cases and four PVL-negative MRSA bacteraemia cases of the same multilocus sequence type (MLST) were isolated during this period (Table 2). The bacteraemia cases were of community onset origin. Three of the patients died.

**Table 1.** MRSA bacteraemia cases and demographic data

MRSA bacteraemia cases	
Total	12
PVL-positive	8
PVL-negative	4
**Demographic data**	
Age range	33–51 years old (median 41 years)
PWID	12 (100 %)
Gender	Male=9, female=3 (total 12)
PVL-positive cases by gender	Male=6, female=2 (total 8)
PVL-negative cases by gender	Male=3, female=1 (total 4)

MRSA, methicillin-resistant *Staphylococcus aureus*; PVL, Panton–Valentine leukocidin; PWID, people who inject drugs.

**Table 2.** Whole-genome sequencing results (WGS) and timeline

Timeline by year	SNP address	PVL genes	SCCmec type
2018	5.2.233.249.272.1742.1991	*luk*PV-F, *luk*PV-S	IV(2B)-c
2018	5.6.328.352.1259.1372.1569	Not detected	IV(2B)-g
2019	5.6.328.352.1259.1906.2201	Not detected	IV(2B)-g
2020	5.2.233.249.272.2237.2609	*luk*PV-F, *luk*PV-S	IV(2B)-c
2020	5.6.328.352.2161.2361.2756	Not detected	IV(2B)-g
2020	5.2.233.249.272.2392.2795	*luk*PV-F, *luk*PV-S	IV(2B)-c
2020	5.2.233.249.272.2516.2943	*luk*PV-F, *luk*PV-S	IV(2B)-c
2020	5.2.233.249.272.2526.2955	*luk*PV-F, *luk*PV-S	IV(2B)-c
2020	5.2.233.249.272.2527.2956	*luk*PV-F, *luk*PV-S	IV(2B)-c
2021	5.6.328.352.2443.2686.3135	Not detected	IV(2B)-g
2022	5.2.233.249.272.3200.3744	*luk*PV-F, *luk*PV-S	IV(2B)-c
2022	5.2.233.249.272.3237.4053	*luk*PV-F, *luk*PV-S	IV(2B)-c

European Nucleotide Archive. Accession number, PRJEB62493.

PVL, Panton–Valentine leukocidin; SCCmec, staphylococcal cassette chromosome mec; SNP, single-nucleotide polymorphism.

Among the eight PVL-positive bacteraemia cases, all eight patients reported skin and soft tissue infections (100 %) (Table 3). There were three cases of pneumonia associated with PVL-MRSA infection. Two patients developed PVL-MRSA bacterial endocarditis. Two patients died and PVL-MRSA bacteraemia was included as a cause of death.

**Table 3.** Clinical summary of MRSA bacteraemia cases

Age and gender summary	Presentation	Comorbidities	PVL toxin	Hospital admission in the 30 days prior to MRSA bacteraemia	Management	Clinical outcome
Age range: 33–51 years old (median 41 years) Gender: 9 male, 3 female	SSTI	PWID, past treated hepatitis C	Positive	No	Incision and drainage, linezolid	Discharged
	SSTI	PWID, bi-polar	Negative	No	Incision and drainage, linezolid	Discharged
	SSTI, staphylococcal pneumonia	PWID, hepatitis C, no fixed abode	Negative	No	Initial vancomycin. Then linezolid and rifampicin. Drainage of para- pneumonic pleural effusion	Discharged
	SSTI, pyelonephritis, peri-nephric collection, staphylococcal pneumonia	PWID, chronic kidney disease, alcohol excess	Positive	No	Initial vancomycin and rifampicin. Then linezolid and rifampicin. Complicated by non-compliance. Drainage of peri-nephric collection	Discharged
	Infective endocarditis, intracerebral haemorrhage, staphylococcal pneumonia	PWID, hepatitis C, no fixed above, DVT, spice misuse	Negative	No	Intensive care admission (intubation and ventilation). Palliation	Died
	SSTI, infective endocarditis, combined enterococcal bacteraemia	PWID, previous infective endocarditis, hepatitis C, pulmonary embolus	Positive	No	Rifampicin, daptomycin and benzyl-penicillin. Then rifampicin and co-trimoxazole. 6 weeks total. Compliance issues	Discharged
	Groin abscess, infective endocarditis, combined group A Streptococcus bacteraemia	PWID, previous infective endocarditis and empyema	Positive	No	Initial clindamycin and teicoplanin. Intensive care admission (enhanced monitoring). Diagnosed with infective endocarditis on echocardiogram. Died shortly after from sepsis	Died
	SSTI, multifocal spinal abscess, osteomyelitis	PWID	Positive	No	Initial teicoplanin and rifampicin then fusidic acid and linezolid. Total 6 weeks. Ultrasound drainage	Discharged
	SSTI, staphylococcal pneumonia, COVID-19, multi-organ failure	PWID, asthma, previous septic arthritis	Positive	No	Initial linezolid, then teicoplanin, rifampicin. Intensive care admission (intubation and ventilation, vasopressors, haemofiltration). Then daptomycin, rifampicin and clindamycin	Died
	Infective endocarditis, staphylococcal pneumonia	PWID, dental caries	Negative	Yes	Vancomycin, linezolid, fusidic acid. Dalbavancin (single dose). Total 6 weeks	Discharged
	Groin abscess	PWID	Positive	No	Incision and drainage	Discharged
	Infected DVT, staphylococcal pneumonia	PWID, groin abscess	Positive	No	Initial vancomycin, rifampicin and linezolid. Then daptomycin, rifampicin and linezolid	Discharged

DVT, deep vein thrombosis; MRSA, methicillin-resistant *Staphylococcus aureus*; PVL, Panton–Valentine leukocidin; PWID, person who injects drugs; SSTI, skin and soft tissue infection.

Of the four related PVL-negative MRSA bacteraemia cases, two patients developed MRSA bacterial endocarditis, and one of these patients died from complications of this MRSA infection. Two patients were treated for skin and soft tissue infections, and one was complicated by a cavitating pneumonia that responded to antimicrobial therapy.

All 12 MRSA isolates were sent to the staphylococcal reference laboratory at Colindale for PVL testing and WGS (Table 2). WGS detected the PVL gene in eight of the MRSA isolates. WGS identified that all isolates were of the same MLST, sequence type 5 (ST5). Genetic relatedness was confirmed by SNP difference, with these isolates belonging to a 10 SNP cluster (range 3–25 SNPs). The PVL- positive isolates carried SCCmec type IV-c and PVL-negative isolates carried SCCmec type IV-g. The isolates were found to be beta-lactam-resistant and additionally carry msrA and/or ermC encoding macrolide resistance.

Comparison of MRSA isolates from Doncaster and Bassetlaw Teaching Hospitals was undertaken with UKHSA with reference to the national reference laboratory WGS archive. This identified further related cases outside of the South Yorkshire area, including Leeds (three cases 2017–2020), Hull (one case in 2021) and Stoke-on-Trent (one case in 2021). Additionally, at time of writing, there have been further Doncaster cases identified amongst PWID. These results are the initial cases that prompted the larger investigation of an ongoing and developing outbreak.

## Discussion

This outbreak of PVL-MRSA highlights the transmissibility, pathogenic potential and severe clinical disease spectrum within the PWID population. Initial public health measures have been undertaken to tackle this outbreak. However, further work is needed to contain this infection and prevent the widespread transmission of this strain across the community.

This strain of PVL-MRSA appears to be associated with severe morbidity and mortality in invasive disease in PWID. It causes a broad range of clinical presentations, including SSTIs, infective endocarditis and cavitating pneumonia. SSTIs in these patients frequently required operative management, which is in line with the published literature [2]. Treating cases of PVL-MRSA bacterial endocarditis proved challenging, as there are no specific treatment guidelines. Management was individualized based on the antibiogram of the isolate and consideration was given to providing adequate anti-PVL toxin cover and was often adapted when faced with intravenous access problems in this challenging cohort of patients. Where faced with difficulties in intravenous access amongst PWID, we had to tailor management to select oral agents with good bioavailability as well as anti-PVL toxin cover, such as linezolid or rifampicin. Although not currently part of UK or European endocarditis guidelines, there is evidence where partial treatment with oral agents in stable patients with bacterial endocarditis (including *S. aureus*) has been proposed [11]. In addition, dalbavancin was used off-label for treatment of MRSA bacteraemia and endocarditis, again due to the challenge of intravenous access and difficulty in monitoring levels of alternative glycopeptides in the PWID population. Given the small numbers of bacteraemia cases presented, it is difficult to comment on how individual antibiotic regimes may have impacted on patient outcomes.

Managing these PVL-MRSA bacteraemia cases has led to a change in our clinical practice when treating PWID presenting with sepsis. Where there is clinical possibility of sepsis from staphylococcal infection, we treat PWID with both anti-MRSA and anti-PVL toxin antibiotics and recommend prompt source control. These cases have highlighted the importance of early screening tests in sepsis, including multiple sets of blood cultures and admission MRSA screening to improve clinical outcomes and prevent onward spread of infection within the healthcare environment.

There is ongoing debate around the severity of infection with PVL-producing strains and the associated clinical outcomes. Initial recognition of PVL-SA infections found PVL-SA causing necrotizing pneumonia and was associated with mortality [12].

However, a systematic review and meta-analysis published in the *Lancet* challenged the view that PVL causes mostly invasive disease associated with a poor prognosis [2]. Infection with a PVL-SA did not predict poor clinical outcome for bacteraemia, musculoskeletal infection and pneumonia. However, PVL-SA SSTIs were more likely to require surgical intervention. The metanalysis excluded many subgroups, including patients in intensive care units and institutions, such as prisons, and concluded that further population-based studies are needed, which adds relevance to the need for this research in the PWID population.

More recent evidence investigating staphylococcal pneumonia in intensive care units has found that infection with PVL-SA is an independent risk factor for mortality in adults and has proposed major differences in pathology of PVL-SA delineated by age [3]. There is a pleuropneumonia in toddlers (<3 years), characterized by pleural effusion and peripheral chest radiograph opacities, and this presentation has been associated with a better clinical outcome. In adults, a necrotizing pneumonia has been described, characterized by airway haemorrhage and rash, preceding flu-like symptoms, and is associated with mortality [3]. The authors suggested that these distinct clinical and radiological entities may have led to underreporting of the severity of PVL-SA pneumonia in previous studies. Amongst PWID, our cases indicate that PVL- MRSA is pathogenic and causes a severe spectrum of clinical disease.

All isolates were MRSA sequence type 5, confirmed by WGS at UKHSA Staphylococcal and Streptococcus Reference Laboratory. PVL-MRSA sequence type 5 has been reported from multiple continents worldwide and has been previously isolated in the UK [13]. The isolates belonged to a 10 SNP cluster (range 3–25 SNPs) over a 4 year period, confirming transmission. The PVL-negative MRSA isolates belong to the Bristol clade (ST5 SCC mec type IV-g), which has been found to infect and colonize PWID, and it is interesting to note the extension of this clone outside the southwest of England [7].

These reported cases are the initial cases isolated at Doncaster and Bassetlaw Teaching Hospitals, which have triggered an in-depth investigation by UKHSA into the PWID community in Doncaster. The outbreak strain has now been identified as causing a fatal community-acquired PVL-MRSA staphylococcal pneumonia in a male patient who was not known to engage in injecting drug misuse in South Yorkshire. Additionally, discussion with UKHSA has led to identification of further related cases outside of the South Yorkshire area in Leeds (three cases), Hull (one case) and Stoke-on-Trent (one case). These patients were of a similar age, but it is unknown whether they identify as PWID.

At time of writing, there have been further cases isolated amongst PWID in Doncaster and UKHSA lead on a case–control study to identify any commonality and lead on the next stage of public health intervention.

Thus far, in response to this outbreak of PVL-MRSA, an initial series of outbreak meetings have been held with UKHSA. Epidemiological factors have been explored, including direct contact at a local sheltered accommodation, needle sharing and the possibility of a contaminated drug supply. The local health protection team has undertaken community screening at a local sheltered accommodation and offered decolonization to screen positive contacts in the community. Additionally, they have provided targeted health education on the prevention of PVL-SA infections at a local substance misuse group. Measures to explore whether the injectable drug supply could be contaminated, and a possible source of the outbreak, has led to identification of a local drug dealer. However, MRSA screening swabs from this person, who was known to hide narcotics on their person, were negative.

Post-infection review has been undertaken in each case by the local clinical commissioning group, which has highlighted the community-acquired nature of these MRSA bacteraemia cases. One patient had had a brief admission to hospital ambulatory care in the 30 days prior to bacteraemia. This episode lasted under 3 h and post-infection review concluded that this was unrelated to subsequent MRSA bacteraemia.

Tackling an organism spread by direct contact presents a major challenge when working with PWID in the community. Patients living with substance misuse disorders may have difficulty engaging with public services and may also engage in needle sharing. Additionally, a number of these patients were of no fixed abode, had high levels of social deprivation and faced significant barriers to engaging in infection prevention measures, such as maintaining personal hygiene, laundering clothes regularly and keeping wounds and abrasions clean. This population is at risk of infection with PVL-SA, with similar outbreaks amongst PWID having been reported previously [7].

In conclusion, these cases of PVL-MRSA infections in PWID highlight the transmissibility, pathogenic potential and severe clinical disease spectrum within this population. PVL- MRSA is of major public health concern and further work is required to tackle this ongoing and developing outbreak and prevent further spread and disease throughout the community.
